# Modeling Heterogeneity in Direct Infectious Disease Transmission in a Compartmental Model

**DOI:** 10.3390/ijerph13030253

**Published:** 2016-02-24

**Authors:** Lingcai Kong, Jinfeng Wang, Weiguo Han, Zhidong Cao

**Affiliations:** 1 State Key Laboratory of Resources and Environmental Information System, Institute of Geographic Sciences and Natural Resources Research, Chinese Academy of Sciences, Beijing 100101, China; konglc@lreis.ac.cn; 2 University of the Chinese Academy of Sciences, Beijing 100049, China; 3 Key Laboratory of Surveillance and Early-Warning on Infectious Disease, Chinese Center for Disease Control and Prevention, Beijing 102206, China; 4 Jiangsu Center for Collaborative Innovation in Geographical Information Resource Development and Application, Nanjing 210023, China; 5 University Corporation for Atmospheric Research/Visiting Scientist Programs, 3090 Center Green Drive, Boulder, CO 80301, USA; hanwg.bj@gmail.com; 6 State Key Laboratory of Management and Control for Complex Systems, Institute of Automation, Chinese Academy of Science, Beijing 100190, China; zhidong.cao@ia.ac.cn

**Keywords:** infectious diseases, mathematical models, homogeneous mixing, heterogeneity, negative binomial distribution

## Abstract

Mathematical models have been used to understand the transmission dynamics of infectious diseases and to assess the impact of intervention strategies. Traditional mathematical models usually assume a homogeneous mixing in the population, which is rarely the case in reality. Here, we construct a new transmission function by using as the probability density function a negative binomial distribution, and we develop a compartmental model using it to model the heterogeneity of contact rates in the population. We explore the transmission dynamics of the developed model using numerical simulations with different parameter settings, which characterize different levels of heterogeneity. The results show that when the reproductive number, $R_{0}$, is larger than one, a low level of heterogeneity results in dynamics similar to those predicted by the homogeneous mixing model. As the level of heterogeneity increases, the dynamics become more different. As a test case, we calibrated the model with the case incidence data for severe acute respiratory syndrome (SARS) in Beijing in 2003, and the estimated parameters demonstrated the effectiveness of the control measures taken during that period.

## 1. Introduction

Mathematical models play an important role in understanding epidemic spread patterns and designing public health intervention measures [1,2,3,4]. The traditional deterministic compartmental models usually assume homogeneous mixing, which means that each individual has the same probability of contact with all of the others in the population [4]. However, there is a growing awareness that this assumption is not the case in reality, because heterogeneity can arise due to many sources [5], including age, sex, susceptibility to disease, position in space and the activities and behaviors of individuals, among others [6]. Here, we will focus on the heterogeneity in host contact rates at the population level.

In recent years, scientists have developed different approaches to model heterogeneity in host contact rates. First, traditional compartmental models were extended: the infection term of the homogeneous mixing compartmental models was modified [7,8,9]. The compartments were further divided into multiple subgroups with similar behavioral characteristics (e.g., risk [10]) or demography (e.g., age [11,12]). Second, along with the rapid development in research on complex networks, a large body of literature has examined the effects of the heterogeneous contact structure on disease spread in networks [13,14]. The third type of modeling approach considering heterogeneity is agent-based modeling [15,16,17], which characterizes the heterogeneity in individual attributes and behaviors. Additionally, several researchers have attempted to bridge the gap between traditional compartmental models and individual-based models [18,19,20].

In this paper, we develop a new compartmental model to incorporate heterogeneous contact rates in disease transmission. First, by combining a Poisson distribution and a Gamma distribution, we derived a negative binomial distribution (NBD) transmission function, with which we developed a compartmental model. Then, we explored the influence of different levels of heterogeneity on the transmission dynamics of infectious diseases using numerical simulations. Finally, we calibrated the model with the number of daily cases of severe acute respiratory syndrome (SARS) in Beijing in 2003, and the estimated parameters show that the control measures taken at that time were effective.

## 2. Methods

### 2.1. NBD Transmission Function

The heterogeneity in transmission can be modeled by assuming that the number of contacts among individuals varies from person to person. Let $X_{i}$ represent the number of effective contacts (the number of contacts that would be sufficient for transmitting the disease successfully, were it to occur between a susceptible individual and an infectious individual [21,22]) with infectious individuals of the *i*-th susceptible person per unit time. Assume that $X_{i}$ has a Poisson distribution $\pi (\theta_{i})$, where $\theta_{i}$ is the mean of the number of effective contacts that the *i*-th susceptible individual makes with infectious individuals per unit time. That $\theta_{i}$ are identical means that each individual has an equal chance of effective contact with infectious individuals and an equal chance of being infected, thereby resulting in a traditional homogeneous-mixing model. In reality, however, individuals typically come into contact with only a small, clustered, subpopulation [20]. Therefore, it is reasonable to assume that different individuals have different average effective numbers of contacts in a certain period of time; that is, $\theta_{i}$ is itself a random variable. The Gamma distribution is a good choice for describing $\theta_{i}$ for a variety of reasons: it is bounded on the left at zero (the numbers of contact must be non-negative), is positively skewed (it has non-zero probability of an extremely high number of contacts) and can represent a variety of distribution shapes [23]. It has been used to describe the expected number of secondary cases caused by a particular infected individual [24]. Therefore, we assume a Gamma distribution for $\theta_{i}$, with shape parameter *k*, rate parameter *m* (or scale parameter $\frac{1}{m}$) and the following probability distribution function:

(1) $$g\left(\theta \right)=\frac{m^{k}}{\Gamma (k)}\theta^{k-1}e^{-m\theta },\;\;\theta >0$$

The conditional distribution of $X_{i}$ given $\theta_{i}=\theta$ is:

$$P\left(X_{i}=x|\theta_{i}=\theta \right)=\frac{e^{-\theta }\theta^{x}}{x!},\;\;x=0,1,2,\cdots$$

We obtain the marginal distribution of $X_{i}$:

P(Xi=x)=∫0∞g(θ)P(Xi=x|θi=θ)dθ=x+k-1x(mm+1)k(1m+1)x,x=0,1,2,⋯

This is the probability density function of an NBD with mean $\frac{k}{m}$ and variance $\frac{k(1+m)}{m^{2}}$. Then, the probability of a susceptible individual escaping from being infected can be represented by the zero term of the NBD:

$$p=P\left(X_{i}=0\right)={\left(\frac{m}{m+1}\right)}^{k}={\left(1+\frac{1}{m}\right)}^{-k}$$

Let the mean of the NBD be equal to the mean of the number of effective contacts of all susceptible individuals with infectious individuals, that is $\frac{k}{m}=\frac{\beta I}{N}$, where *β* denotes the transmission rate, defined as the per capita rate at which two specific individuals come into effective contact per unit time [22]; *I* denotes the number of infectious individuals; and *N* denotes the size of the total population. It follows that $\frac{1}{m}=\frac{\beta I}{kN}$, and:

$$p={\left(1+\frac{\beta I}{kN}\right)}^{-k}$$

Consider a closed population (without births, deaths and migration into or out of the population). Let $S_{t}$ and $I_{t}$ denote the numbers of the susceptible and infectious individuals at time *t*, respectively. Then, the difference equation relating $S_{t}$ and $I_{t}$ at successive time steps *t* and t+1 is:

$$S_{t+1}=S_{t}{\left(1+\frac{\beta I_{t}}{kN}\right)}^{-k}=S_{t}-\left[1-{\left(1+\frac{\beta I_{t}}{kN}\right)}^{-k}\right]S_{t}$$

Here, $\lambda_{t}=1-{\left(1+\frac{\beta I_{t}}{kN}\right)}^{-k}$ is the risk of a susceptible individual becoming infected between time *t* and t+1. Using the relationship between the risk and rate derived in [22], $risk=1-e^{rate}$, we obtain the rate at which susceptible individuals become infected at time *t*:

$$\lambda \left(t\right)=k\ln\left(1+\frac{\beta I}{kN}\right)$$

Therefore, the rate of change in the number of susceptible individuals can be represented by the differential equation representing:

$$\frac{dS}{dt}=-k\ln\left(1+\frac{\beta I}{kN}\right)S$$

We call $k\ln(1+\frac{\beta I}{kN})$ in the right side of this equation the NBD transmission function. A similar function, $k\ln(1+\frac{aP_{t}}{k})$, and its discrete form, ${\left(1+\frac{aP_{t}}{k}\right)}^{-k}$, were first used in host-parasitoid models, where *a* denotes the per capita searching efficiency of the parasitoid and $P_{t}$ denotes the number of parasitoids [25,26]. Then, they were used in insect-pathogen models [27]. In [28], the author used the transmission function, $k\ln(1+\frac{\beta I}{k})$, to model a possum-tuberculosis (TB) system. The influence of different transmission functions on a simulated pathogen spread was studied in [29]. Because:

(2) $$\lim_{k\to \infty }k\ln\left(1+\frac{\beta I}{kN}\right)S=\frac{\beta SI}{N}$$

(3) $$\lim_{k\to \infty }k\ln\left(1+\frac{\beta I}{k}\right)S=\beta SI$$

when k→∞, the NBD transmission function we derived here approximates the frequency-dependent transmission function of the homogeneous-mixing model. Therefore, it can be regarded as a generalized frequency-dependent transmission function [1,4]. Similarly, the NBD transmission function used in [28] can be regarded as a generalized density-dependent transmission function [1,4].

Comparing the NBD transmission function with the density-dependent transmission function, βSI, and the frequency-dependent transmission function, $\frac{\beta SI}{N}$, of the homogeneous-mixing model [4,22], we obtain one more parameter, *k*, which is the shape parameter of the Gamma distribution (Equation (1)). Denote the mean of the Gamma distribution as $\mu_{\theta }$; then, the variance is $\frac{\mu_{\theta }^{2}}{k}$. Setting the mean to be a constant and letting k→∞, the variance goes to zero, resulting in homogeneous-mixing, just as shown in Equation (2). In contrast, the variance increases as the value of *k* decreases, which indicates greater heterogeneity of the contact rates between the susceptible and infectious populations. Therefore, the parameter *k* characterizes the level of heterogeneity.

### 2.2. NBD Compartmental Model

**Figure 1:**
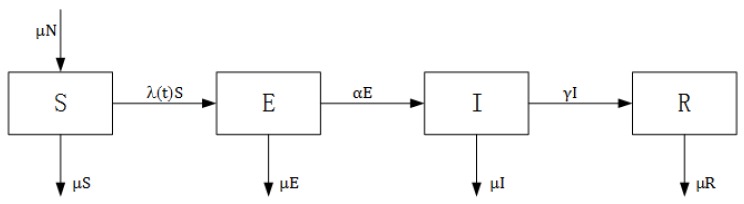
Structure of a susceptible-exposed-infectious-recovered (SEIR) model.

The standard susceptible-exposed-infectious-recovered (SEIR) model divides the total population into four compartments: susceptible (S, previously unexposed to the pathogen), exposed (E, infected, but not yet infectious), infected (I, infected and infectious) and recovered (R, recovered from infection and acquired lifelong immunity) [1,4,22]. The infection process is represented in Figure 1. Children are born susceptible to the disease and enter the compartment S. A susceptible individual in compartment S is infected after effective contact with an infectious individual in compartment I and then enters the exposed compartment E. After the latent period ends, the individual enters the compartment I and becomes capable of transmitting the infection. When the infectious period ends, the individual enters the recovered class R and will never be infected again [4,22]. In each compartment, individual death occurs at a constant rate, *μ*, which is equal to the birth rate. Death induced by the disease is not considered here. Therefore, the total population size in the model, *N*, remains unchanged. The SEIR model and its extension have been used to model many infectious diseases, for example, measles [30,31,32], rubella [33,34], influenza [35,36] and SARS [37,38], among others.

Using the NBD transmission function, we set up a new SEIR model in a closed population, represented by a set of ordinary differential equations:

(4) $$\left\{\begin{array}{cc} \frac{dS}{dt} & =\mu N-k\ln(1+\frac{\beta I}{kN})S-\mu S\\ \frac{dE}{dt} & =k\ln\left(1+\frac{\beta I}{kN}\right)S-\left(\alpha +\mu \right)E\\ \frac{dI}{dt} & =\alpha E-(\gamma +\mu )I\\ \frac{dR}{dt} & =\gamma I-\mu R \end{array}\right.$$

where the parameter *α* is the rate at which individuals in the exposed category become infectious per unit time, and its reciprocal is the average latent period [4,22]; the parameter *γ* is the rate at which infectious individuals recover (become immune) per unit time, and its reciprocal is the average infectious period [4,22]; and the parameter *μ* refers to the birth and death rates.

Based on the next-generation matrix approach [39], we derive the basic reproductive number (see Appendix **A** for further details),

(5) $$R_{0}=\frac{\alpha \beta }{\left(\alpha +\mu )(\gamma +\mu \right)}$$

which is identical to that of the homogeneous-mixing model with a frequency-dependent transmission function [4]. It is worth noting that it is irrelevant to *k*, which means that it does not depend on the level of heterogeneity. This can be explained by $R_{0}$ being an average quantity, which means that it does not consider the individual variance in infectiousness [24]. This result is in agreement with the conclusion made using a meta population version of the standard stochastic SIR model incorporating spatial heterogeneity [40].

We now determine the equilibrium states. Without much work, we can obtain the disease-free equilibrium (N,0,0,0). We also derive the approximate size of the infectious compartment at the endemic equilibrium, $I^{*}\approx \frac{\mu N}{\beta }\left(R_{0}-1\right)$ when $R_{0}>1$ (Appendix **B**). This is identical to that of the homogeneous-mixing model with a frequency-dependent transmission function [4]. Similar to $R_{0}$, it does not depend on *k*. In other words, the contact heterogeneity does not influence the endemic equilibrium, although it does change the dynamics, which we demonstrate using numerical simulations in the next section.

## 3. Results

### 3.1. Dynamics of the NBD Model

**Figure 2:**
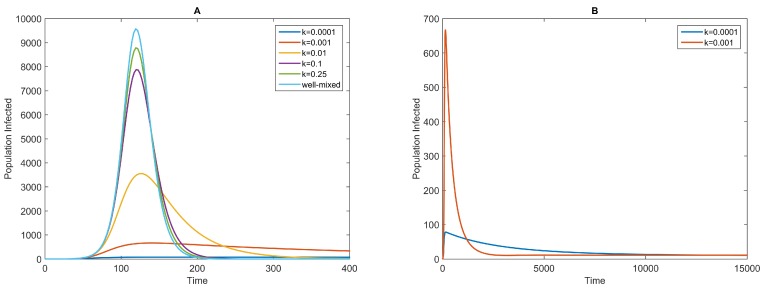
Infectious curves for different values of *k* and fixed *β* for the negative binomial distribution (NBD) model (Equation (4)). The values of *k* are shown in the legend. The other parameters are as follows: β=0.5, $\frac{1}{\alpha }=7$ days, $\frac{1}{\gamma }=5$ days and $\frac{1}{\mu }=70$ years. The initial conditions are S(0)=99,999,E(0)=0,I(0)=1 and R(0)=0. The top curve in (**A**) is the infectious curve of the homogeneous-mixing model with a frequency-dependent transmission term [4]; it is compared to the infectious curves of the NBD model; (**B**) The long trend of the infectious curves of the NBD model with k=0.0001 and k=0.001.

Using numerical simulations, we explore the influence of the heterogeneity level on the transmission dynamics, characterized by the parameter *k*. The results show that the infectious curves with fixed *β*, but different values of *k* achieve a peak after a period that is almost the same in duration (Figure 2A). However, the transmission speed and, therefore, the peak size, as well as the dynamics after the peak are very different. A low level of heterogeneity results in dynamics similar to those predicted by the homogeneous-mixing model with a frequency-dependent transmission term, $\frac{\beta SI}{N}$. This is consistent with the conclusion inferred in Equation (2).

As the value of *k* decreases, that is the level of heterogeneity increases, the dynamics differ increasingly from those predicted by the homogeneous-mixing model. The greatest difference is that at the overall level, the heterogeneity slows the transmission speed and decreases the peak sizes, which means milder disease outbreaks, because in the scenario with a high level of heterogeneity, only a small proportion of susceptible individuals have chances of coming into contact with infectious individuals and becoming infected, which results in a slower increase of the infected population. Second, after the peak is attained, the infectious curves do not decline as rapidly as those predicted by the homogeneous-mixing model and the NBD models (Equation (4)) with larger values of *k* (Figure 2A), and the disease persists over a long term in the population (Figure 2B). Compared to the homogeneous-mixing model or the NBD models with larger values of *k*, up to the peak time (almost the same), there are many more individuals who are still susceptible to the disease. A proportion of them come into contact with infectious individuals and become infected, and this process persists for a long period of time. Moreover, Figure 2B shows that the endemic sizes of the two scenarios are approximately equal, just as noted in the previous section. In addition, when *k* drops to a very small value, there will be no disease outbreak, because almost none of the susceptible individuals have any chance of coming into contact with infectious individuals and becoming infected. It is shown that the contact patterns exhibit more heterogeneity than that assumed by homogeneous-mixing models, but they do not appear extremely heterogeneous [6].

**Figure 3:**
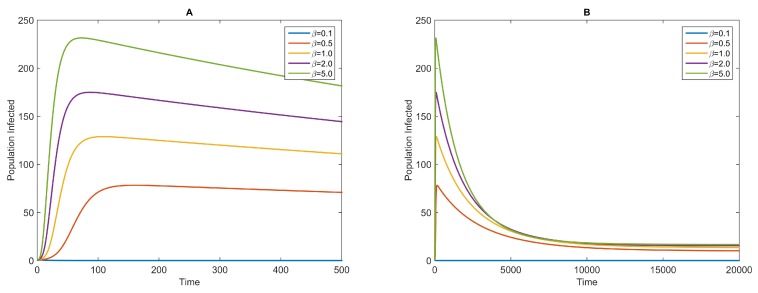
Infectious curves for different values of *β* and fixed *k* for the NBD model (Equation (4)). The values of *β* are shown in the legend. The other parameters are as follows: $k=10^{-4}$, $\frac{1}{\alpha }=7$ days, $\frac{1}{\gamma }=5$ days and $\frac{1}{\mu }=70$ years. The initial conditions are S(0)=99,999,E(0)=0,I(0)=1 and R(0)=0. (**A**) The infectious curves around the peak; (**B**) The long trend of the infectious curves of the NBD model with the same parameters.

We also simulate the dynamics with a fixed value of *k* and different values of *β*. Because the dynamics obtained with a large value of *k* are similar to those of the homogeneous-mixing model with a frequency-dependent transmission term, we only show the results for a relatively small value of $k=10^{-4}$ (Figure 3). For larger values of *β*, the infectious curves reach their peaks earlier, and the peaks are higher than those obtained for smaller values of *β*. After the peak of the disease outbreak is achieved, the infectious curves decrease slowly and reach endemic equilibrium gradually (Figure 3B). Additionally, for much smaller values of *β*, such that $R_{0}<1$, there will be no disease outbreak (here, for example, β=0.1).

### 3.2. Fitting the NBD Model to the 2003 Beijing SARS Outbreak Data

**Figure 4:**
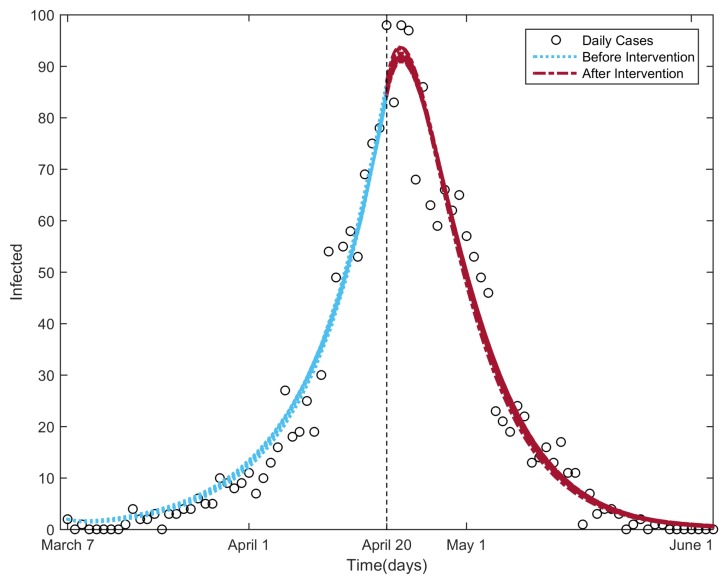
Infectious curves for the fitting procedure of the NBD model to the SARS outbreak in Beijing in 2003. The circle markers denote the daily reported SARS cases; the parts of the curve to the left and right of the vertical line are the infectious curves before and after the control strategies were taken, respectively.

The SARS disease broke out in the beginning of March 2003 in Beijing, spread rapidly over the next six weeks and peaked during the third and fourth weeks of April [41]. In total, 2048 confirmed cases were reported during the entire outbreak period (the circle markers shown in Figure 4; the data were provided by the Chinese Center for Disease Control and Prevention). Prompted by the rapid expansion of the epidemic, on 17 April, the Beijing municipal government established a Joint SARS Leading Group and deployed 10 task forces to oversee crisis management [41,42]. On 20 April, a larger number of cases was reported, and the Chinese government canceled the May Day holiday in an effort to reduce the mass movement of people [43]. Multiple measures were taken to control the spread of the disease, including the provision of personal protective equipment and training for healthcare workers [41]; introduction of community-based prevention and control through case detection, isolation, quarantine and community mobilization [41]; closure of the sites of public entertainment and schools [42]; and stopping the entry of all visitors or screening them for fever upon entry to universities and other places [42]. Additionally, a general increase in SARS awareness played an important role in controlling the outbreak [42]. The multiple measures implemented in Beijing likely led to the rapid resolution of the SARS outbreak [42].

To evaluate the effectiveness of the control measures taken in Beijing at that time, we calibrated the NBD model to the data of the SARS daily cases using the GlobalSearch algorithm in the MATLAB Global Optimization Toolbox [44,45] and estimated the parameters. We used two different values, $k_{1}$ and $k_{2}$, to characterize the different levels of heterogeneity in contact in the population before and after 20 April [38]. We assumed a fixed value for *β* for simplicity (in reality, the value of *β* decreased along with the control strategies [38]; we mainly discuss the influence of the other parameter, *k*). We chose the normalized root mean square error (NRMSE) [46] as the goodness of fit between the model output and the daily case data, as well as the objective function of the calibration procedure. In order to compute the NRMSE, we solved the set of differential equations (Equation (4)) with unknown parameters α,β,γ and $k=k_{1}$ from 7 March to 20 April. The initial conditions were set as follows: $S\left(0\right)=1.4564\times 10^{7}$, which was the size of the permanent population in Beijing in 2003 [47]; t=0 corresponds to 7 March 2003; E(0)=0; I(0)=2, which was the number of daily cases on 7 March 2003; and R(0)=0. Then, the output of the model on 20 April was taken as the initial value to solve Equation (4) with parameters α,β,γ and $k=k_{2}$ from 20 April to 4 June. Finally, the two outputs were combined and used to calculate the goodness of fit to the SARS daily case data. The birth and death rate, *μ*, was assumed to be 1/70 $year^{-1}$. In total, there were five unknown parameters to be estimated: $k_{1},k_{2},\alpha ,\beta$ and *γ*.

The starting points of the parameters for the calibration procedure were selected randomly between the bounds of the parameters shown in Table 1.

Because of the stochasticity of the GlobalSearchalgorithm [44,45], the results varied slightly every time. We ran the procedure 100 times. Table 2 presents the minimum, maximum, mean and standard variance of the results. The average latent and infectious periods are $\frac{1}{\alpha }=6.8661$ days and $\frac{1}{\gamma }=4.8439$ days, respectively. The much smaller $k_{2}$ value indicates that the control measures are extremely effective in controlling the SARS transmission in Beijing in 2003. This is in agreement with the result in [38]. Figure 4 shows the 100 fitted infectious curves and the daily cases.

## 4. Discussion

In this paper, we aimed to study the influence of heterogeneity in the contact rates in disease transmission at the population level. The developed NBD model can be regarded as a generalized homogeneous-mixing model with a frequency-dependent transmission function. Our results show that, keeping other conditions identical, the higher is the level of heterogeneity in contact rates, the greater is the difference in the disease dynamics observed from those predicted using the homogeneous-mixing models.

It is worthwhile to compare our approach and results to previous approaches and results. To address heterogeneous-mixing within populations, the populations were further divided into multiple subgroups [10,11,12], and used the WAIFW matrix (“who acquires infection from whom” [1]), in which any individual is more likely to come into contact with other individuals from within the same subgroup than those outside. However, in this framework, contact rates within the subgroups are still homogeneous. A different class of approaches for extending the traditional compartmental models to incorporate heterogeneity involves modifying the transmission term; our approach belongs to this class. The work in [7,8,19] replaced the bilinear transmission term (SI) in the homogeneous compartmental model with a nonlinear term $kS^{p}I^{q}$, where k,p,q are the “heterogeneity parameters”. Their results showed that the modified model was capable of predicting the disease transmission patterns in a clustered network [19]. Stroud *et al.* used a power-law scaling of the new infection rate $I{\left(S/N\right)}^{v}$, with scaling power *v* greater than one, to relax the homogeneous-mixing assumption [9], and it was demonstrated that this power-law formulation leads to significantly lower predictions of the final epidemic size than the traditional linear formulation. Compared to these empirical or semi-empirical modifications [7,8,9,19], the NBD transmission function seems to agree more with the real transmission mechanics, in that it assumes that the mean of the number of effective contacts of the susceptible individuals with infectious individuals per unit time is different from individual to individual, and the choice of the Gamma distribution offers multiple advantages (see Section 2.1).

In recent years, several network-based models have been developed to study the influence of contact heterogeneity on disease transmission. Keeling *et al.* reviewed multiple types of networks and the statistical and analytical approaches for the spread of infectious diseases [13,14]. In particular, Bansal *et al.* demonstrated that the high-level heterogeneous degree distributions generate an almost immediate expansion phase compared to homogeneous degree distributions, such as the Poisson distribution [6,49,50]. The NBD-SEIR model does not exhibit this feature. We suspect that this is because our approach belongs to the mean-field class of approaches and considers a large population at the overall level. In addition, it is possible to approximate the main features of disease spread in networks with compartmental models using an appropriate construction. The work in [20] used $R_{0}$ as a fundamental parameter to formulate a mean-field type model, which can implicitly capture some important effects of heterogeneous-mixing in contact networks. The work in [51,52] applied “edge-based compartmental modeling” (EBCM), which focuses on the status of a random partner rather than a random individual, to capture the heterogeneous contact rates in disease transmission.

Although it incorporates the heterogeneous contact rates in disease transmission in a tractable manner, the NBD model has some weaknesses. First, the parameter *k* characterizes the level of heterogeneity, which is difficult to measure directly, and this can be overcome by using contact tracing data. Second, some features cannot be recovered by the NBD model. In future research, it will be interesting to incorporate other factors that influence transmission dynamics, such as the migration of populations, seasonality and vaccinations, among others.

## 5. Conclusions

Using the probability density function for the negative binomial distribution, we constructed a NBD transmission function and further developed a compartmental model for direct infectious disease. The developed model considers the heterogeneity of contact rates in the population. The simulation results show that, at the population level, the dynamics vary widely according to the level of heterogeneity in contact rates. Once $R_{0}>1$, a low level of heterogeneity results in dynamics similar to those predicted by the homogeneous mixing models. Keeping other conditions identical, as the level of heterogeneity increases, the transmission speed becomes more and more slowly, the peak size becomes smaller and smaller. These results have implications for developing interventions, such as isolation, targeted vaccination, among others.

## Figures and Tables

**Table 1:** Parameter notations, biological meanings, values and sources.

Parameter	Biological Meaning	Bound/Value	Source
$k_{1}$	Heterogeneity level before intervention	$\left(10^{-12},10^{-4}\right)$	Assumed
$k_{2}$	Heterogeneity level after intervention	$\left(10^{-12},10^{-4}\right)$	Assumed
*β*	Transmission rate	(0.1,10)	Assumed
1/α	Latent period	(2,7) days	[48]
1/γ	Infectious period	(2,10) days	[48]
1/μ	Expected human lifetime	70 years	Assumed

**Table 2:** Descriptive statistics of the fitted parameters.

Parameter	Minimum	Maximum	Mean	Standard Variance
$k_{1}$	$8.4123\times 10^{-6}$	$6.1781\times 10^{-5}$	$1.1882\times 10^{-5}$	$5.75\times 10^{-6}$
$k_{2}$	$1.0130\times 10^{-12}$	$1.1585\times 10^{-9}$	$2.6311\times 10^{-11}$	$1.4077\times 10^{-10}$
*β*	0.3525	0.6109	0.5459	0.0335
*α*	0.1429	0.2130	0.1456	0.0095
*γ*	0.1407	0.2366	0.2064	0.0118
NRMSE	0.8005	0.8041	0.8037	$7.2604\times 10^{-4}$

## Appendix

### Appendix A. R 0 Expression for the Model

Using the next-generation operator approach [39], we compute the basic reproductive number $R_{0}$. First, we sort the compartments so that the first *m* compartments correspond to infected individuals: x=(E,I,S,R). Here, the infected compartments are E and I, yielding m=2. Then, we decompose the components of the differential equations into F, in which $F_{i}$ is the rate of appearance of new infections in compartment *i*, and V, in which $V_{i}$ is the rate of transfer of individuals into and out of compartment *i* by all other means:

$$F=\left(\begin{array}{c} k\ln(1+\frac{\beta I}{kN})S\\ 0\\ 0\\ 0 \end{array}\right),\;V=\left(\begin{array}{c} (\alpha +\mu )E\\ (\gamma +\mu )I-\alpha E\\ -\mu N+k\ln(1+\frac{\beta I}{kN})S+\mu S\\ -\gamma I+\mu R \end{array}\right)$$

The disease-free equilibrium (DFE) for this model is $x_{0}=\left(0,0,N,0\right)$. Then,

$$F=\left[\frac{\partial F_{i}}{\partial x_{j}}\left(x_{0}\right)\right]=\left(\begin{array}{cc} 0 & \beta \\ 0 & 0 \end{array}\right),\;V=\left[\frac{\partial V_{i}}{\partial x_{j}}\left(x_{0}\right)\right]=\left(\begin{array}{cc} \alpha +\mu & 0\\ -\alpha & \gamma +\mu \end{array}\right),\;1\leq i,j\leq m$$

giving:

$$FV^{-1}=\left(\begin{array}{cc} \frac{\alpha \beta }{\left(\alpha +\mu )(\gamma +\mu \right)} & \frac{1}{\gamma +\mu }\\ 0 & 0 \end{array}\right)$$

This is called the next-generation matrix for the model [39]. Finally, the basic reproductive number, $R_{0}$, is calculated using the spectral ratio:

$$R_{0}=\rho \left(FV^{-1}\right)=\frac{\alpha \beta }{\left(\alpha +\mu )(\gamma +\mu \right)}$$

### Appendix B. Endemic Equilibrium

Because the total population size *N* is a constant and R=N-S-E-I, the last equation in Equation (4) is redundant. To find the endemic equilibrium, we set the right side of the other three equations to zero. Then, *S* and *E* can be represented by *I*:

$$S=\frac{\mu N}{\mu +k\ln(1+\frac{\beta I}{kN})},\;E=\frac{\gamma +\mu }{\alpha }$$

Substituting them into $k\ln\left(1+\frac{\beta I}{kN}\right)S-\left(\alpha +\mu \right)E=0$ and after some algebraic manipulation, we obtain:

$$\alpha \mu k\ln\left(1+\frac{\beta I}{kN}\right)N-\left(\alpha +\mu \right)\left(\gamma +\mu \right)\left[\mu +k\ln\left(1+\frac{\beta I}{kN}\right)\right]I=0$$

Obviously, it is difficult and even impossible to find an explicit solution. We find an approximate solution using the first-degree Taylor polynomial of ln(1+x) near x=0, that is ln(1+x)≈x. It follows that,

$$\alpha \mu \beta I-\left(\alpha +\mu \right)\left(\gamma +\mu \right)\left(\mu +\frac{\beta I}{N}\right)I\approx 0$$

We obtain the approximate solution for I:

$$I^{*}\approx \frac{\mu N}{\beta }\left(R_{0}-1\right)$$

where $R_{0}$ is given in Equation (5).
